# Comparative transcriptome analysis reveals molecular mechanisms of resistance in Chinese cabbage to *Plasmodiophora brassicae* pathotype 11

**DOI:** 10.3389/fmicb.2025.1495243

**Published:** 2025-01-24

**Authors:** Yue Qiu, Jinhao Zhang, Chunju Deng, Jiasheng Yuan, Bowen Wang, Han Meng, Mohamed Mohany, Liting Zeng, Lanfang Wei, Waqar Ahmed, Guanghai Ji

**Affiliations:** ^1^State Key Laboratory for Conservation and Utilization of Bio-Resources in Yunnan, Yunnan Agricultural University, Kunming, China; ^2^College of Agriculture, Anshun University, Anshun, China; ^3^Key Laboratory of Agro-Biodiversity and Pest Management of Ministry of Education, Yunnan Agricultural University, Kunming, China; ^4^Department of Pharmacology and Toxicology, College of Pharmacy, King Saud University, Riyadh, Saudi Arabia; ^5^Agricultural Foundation Experiment Teaching Center, Yunnan Agricultural University, Kunming, China; ^6^Guangdong Province Key Laboratory of Microbial Signals and Disease Control, College of Plant Protection, South China Agricultural University, Guangzhou, China

**Keywords:** *Plasmodiophora brassicae*, Chinese cabbage, clubroot resistance, transcriptome analysis, defense mechanisms, physiological race

## Abstract

**Background and aims:**

Clubroot caused by the soilborne obligate parasite *Plasmodiophora brassicae*, is a devastating disease of Chinese cabbage and other crucifers. The innate diversity and adaptability of this pathogen pose significant challenges to effective control measures. However, the varied response mechanisms exhibited by hosts to pathotype 11 at a molecular level are still unclear.

**Methods and results:**

This study investigated the resistance response and underlying molecular mechanism of two Chinese cabbage (*Brassica rapa*) varieties (JP and 83-1) to *P. brassicae* pathotype 11 through comparative transcriptome analysis and microscopic study. Results demonstrated that 14 days after inoculation (dai) is a critical time point of the infection process for resistant variety to inhibit the proliferation of *P. brassica*. Although the highly resistant variety JP did not exhibit a complete immune response to pathotype 11, it demonstrated a significant resistance level against *P. brassicae* pathotype 11 by restricting its proliferation in the xylem vessels. Microscopic analysis at 21 dai revealed that the resistant cultivar (JP) root structure remained largely unaffected, while the roots of the susceptible cultivar (83-1) exhibited significant tissue distortion and gall formation, underscoring the effectiveness of the resistance mechanisms. Comparative transcriptome analysis revealed substantial differences in the number and types of differentially expressed genes (DEGs) between the two cultivars, highlighting the key pathways involved in the resistance response. In the resistant cultivar (JP), a total of 9,433 DEGs were identified, with 4,211 up-regulated and 5,222 down-regulated. In contrast, the susceptible cultivar (83-1) exhibited 6,456 DEGs, with 2,781 up-regulated and 3,675 down-regulated. The resistant cultivar showed a pronounced activation of genes involved in hormone signaling, cell wall, secondary metabolism, redox state, and signaling process. Therefore, our speculation revolves around the potential resistant mechanism of this variety, which inhibits the proliferation of *P. brassicae* in the roots via secondary metabolites, cell wall, and ROS and also regulates physiological mechanisms mediated by plant hormones such as ABA to adapt to adverse environmental conditions such as water scarcity induced by the pathogen.

**Conclusion:**

This study unveils the intricate defense mechanisms potentially activated within Chinese cabbage when confronted with *P. brassicae* pathotype 11, offering valuable insights for breeding programs and the development of novel strategies for managing clubroot disease in Brassica crops. Furthermore, this study highlights the pivotal role of host-specific molecular defense mechanisms that underlie resistance to *P. brassicae* pathotype 11.

## 1 Introduction

Chinese cabbage (*Brassica rapa* subsp. *pekinensis*) is an economically important vegetable crop worldwide, including in China (Chai et al., 2014). However, clubroot disease, caused by the obligate parasite *Plasmodiophora brassicae*, is one of the most devastating diseases to Chinese cabbage and other crucifers, leading to substantial yield losses estimated at 10 to 15% worldwide (Peng et al., 2016; Zhou et al., 2022). The pathogen has been identified as a soilborne pathogen with a multifaceted life cycle consisting of three distinct stages: (1) resting spores exist in the soil, (2) the primary infection phase occurs in the root hairs, leads to form secondary zoospores, which are released into the soil and causes secondary infection, (3) the secondary infection phase developed within the cortex cells, resulting in a typical symptom of root galls or clubroots (Liu et al., 2020). *P. brassicae* can survive in the soil for several years as a dormant resting spore without any host or external stimulants, thus making this disease very difficult to control in the field once the soil is contaminated (Vañó et al., 2023).

At present, strategies for preventing and controlling clubroot disease include long-time crop rotation and application of soil amendments (lime and ammonium bicarbonate), fungicides, and biological agents (Zhang et al., 2023; Yang X. et al., 2024). However, while applying these strategies to large-scale field crops is the greatest challenge, some methods are time-consuming, labor-intensive, costly, and impractical (Strelkov et al., 2016; Struck et al., 2022). More importantly, these strategies can only reduce the severity of clubroot disease and fall short of completely eradicating the resting spore of *P. brassicae* from the infested field (Hasan et al., 2021). Cultivating resistant varieties is considered one of the most influential and economical ways to reduce crop yield losses caused by *P. brassicae* infection. The greatest challenge to disease-resistant breeding is the diversity and adaptability of *P. brassicae*. According to the Williams identification system, at least 8 physiological races (1, 2, 4, 7, 9, 10, 11, and 13) have been identified in China (Ning et al., 2018). The introduction of a single resistant variety has brought a shift in the virulence dynamic of *P. brassicae* and, potentially leading to an eventual break through the host’s resistance (Holtz et al., 2018; Strelkov et al., 2018). The best breeding strategy is based on a better understanding of the mechanisms of pathogenesis. Therefore, it is necessary to understand the interaction mechanisms between various physiological races of *P. brassicae* and their hosts.

Transcriptome profiling is a valuable tool for providing global gene expression and exploring the molecular basis of interactions between host and pathogen (Liu et al., 2022). Several RNA-seq-based studies have been conducted in *B. rapa* to elucidate the potential molecular mechanism by mining the key gene responses to the infection of *P. brassicae*. Chen J. et al. (2016) found that after inoculation of *P. brassicae*, most of the DEGs between two near-isogenic lines (NILs) of Chinese cabbage were involved in metabolism, transportation, signal transduction, and defense pathways, and analysis suggests that SA signaling pathway was necessary to clubroot resistance in CR BJN3-2 (Chen J. et al., 2016). Yuan and colleagues identified 10 hub genes highly associated with clubroot resistance through transcriptome and Weighted Gene Co-expression Network Analysis (WGCNA) (Yuan et al., 2021). A study based on transcriptome analysis of *B. rapa* confirmed that genes for auxin signaling were significantly up-regulated during the clubroot development (Robin et al., 2020). Nevertheless, numerous available transcriptomic studies in Cruciferae plants, including *Arabidopsis thaliana*, have predominantly focused on pathotype four or other variants of *P. brassicae,* while there is a limited investigation on pathotype 11 (Irani et al., 2018; Ning et al., 2019; Yuan et al., 2021).

In our previous work, we collected 23 samples of clubroot from various parts of Yunnan Province, China, and used the Williams differential set to identify the physiological race. Among these samples, 15 were identified as race 11, accounting for 65.22%. Subsequently, we selected a highly resistant variety (JP) and a highly susceptible variety (83-1) after screening 67 cabbage-cultivated varieties based on pathotype 11. In this study, we conducted microscopic and transcriptome analysis on the roots of two Chinese cabbage varieties, JP and 83-1, following inoculation with *P. brassicae* pathotype 11. The primary objective of this study is to investigate the resistance mechanisms of two Chinese cabbage varieties, JP and 83-1, against *P. brassicae* pathotype 11. This research aims, (1) to elucidate the histopathological differences observed in root hair and cortical infections between the resistant (JP) and susceptible (83-1) cultivars; (2) to analyze the differences in the transcriptional response of JP and 83-1 to *P. brassicae* pathotype 11 infections; and (3) to elucidate the specific molecular mechanisms underlying resistance in the JP variety, mainly focusing on pathways related to hormone signaling, secondary metabolite production, cell wall fortification, and reactive oxygen species (ROS) regulation. We hypothesize that the findings of our study will provide valuable insights for breeding programs aimed at developing new Chinese cabbage varieties with enhanced resistance to clubroot disease, thereby contributing to sustainable agricultural practices.

## 2 Materials and methods

### 2.1 Plant material and growth conditions

Two Chinese cabbage cultivars with different resistance responses to clubroot pathogen *P. brassicae* pathotype 11 were used in this study as plant material. The Chinese cabbage variety JP is resistant (R) to physiological pathotype 11, while 83-1 is susceptible (S) to the same race. The seeds were sterilized by immersing them in 70% ethanol for 1 min, followed by three times rinses with sterile water. Afterwards, the seeds were planted in plastic trays filled with sterile nursery medium [soil, quartz sand, and vermiculite (1:1:1)] for 3 weeks (Zhang et al., 2024).

### 2.2 Preparation of *Plasmodiophora brassicae* spore suspension

The resting spores of *P. brassicae* were obtained from clubroot pathogen-infected root galls collected from Tonghai City, Yunnan province, China, and stored at −20°C until further use. This pathogen was identified as pathotype 11 based on the Williams differential system. *P. brassicae* spores suspensions were prepared following the methodology of Zhang and colleagues (Zhang et al., 2022) and adjusted to a final spores concentration of 1 × 10^7^ mL^−1^ by a hemocytometer.

### 2.3 Pot experiment and analysis of disease index

In this study, Chinese cabbage cultivars JP (R) and 83-1 (S) were divided into two groups (treatment and control). Six plants were placed in pots (24 cm × 18 cm) filled with 3 kg of disease-free soil, and pots were kept in a growth room at 25°C with a 16/8 h light/dark cycle (Zhang et al., 2022). The soil was kept moist throughout the treatment period by adding 500 mL of water twice a week. Seedlings were allowed to acclimate in these pots for 3 days before inoculating *P. brassicae* spore suspension (1 × 10^7^ mL^−1^). The treatment group was inoculated with 20 mL/pot of *P. brassicae* pathotype 11 spore suspension (1 × 10^7^ mL^−1^) and marked R_Pb and S_Pb, respectively. The control groups were inoculated with 20 mL/pot of sterile water and marked R_CK and S_CK, respectively. The experiment was performed under a completely randomized design and repeated thrice with five pots per treatment served as replicates. Disease index and incidence were recorded and calculated 35 days after transplantation using a 6-point (0, 1, 3, 5, 7, and 9) disease rating scale and formula described by Wei L. et al. (2021).

### 2.4 Root samples collection

Three plants per replication from each treatment were randomly uprooted at 5, 8, 11, 14, and 21 days after inoculation (dai) and shaken gently to remove the excess soil. The roots were then rinsed 5 times with sterilized distilled water to eliminate debris and contamination (Ali et al., 2023). The surface moisture was absorbed with sterilized filter paper, and the sampled roots were immediately frozen in liquid nitrogen and stored at −80°C for subsequent microscopy and transcriptome analyses.

### 2.5 Fluorescence microscope analysis

The *P. brassicae* infection process was observed in the roots of Chinese cabbages collected at 5, 8, 11, and 14 dai under a fluorescence microscope (Leica DM2000, Germany). For sample preparation, segments were cut from lateral roots, stained with 0.5% Phloxine B for 10 min, covered with a coverslip, and observed under the fluorescence microscope (Down, 2000). The effect of *P. brassicae* infection on the host root structure was observed at 21 dai using a differential-interference microscope (BX53, OLYMPUS, Japan). Root samples were prepared for microscope analysis according to Zhao and colleague’s methodology (Zhao et al., 2019). The root samples were first placed overnight at 4°C in a 2.5% glutaraldehyde solution, followed by three times washing with phosphate buffer (0.1 M, pH 7.0)for 15 min each time and then fixed with a 1% osmium acid solution for 1–2 h. After carefully removing the osmic acid liquid, the sample was rinsed thrice with phosphate buffer (0.1 M, pH 7.0)for 15 min each time. The samples were dehydrated using ethanol solutions with gradient concentrations (30, 50, 70, 80, 90, and 95%) for 15 min at each concentration, followed by treatment with 100% ethanol for 20 min. Finally, the samples were transitioned to pure acetone treatment for 20 min. The samples were then treated with a mixture of Spurr embedding agent and acetone (V/V = 1/1) for 1 h, followed by a mixture of Spurr embedding agent and acetone (V/V = 3/1) for 3 h. The samples were then treated at room temperature with pure embedding agent overnight. After penetration treatment and heating overnight at 70°C, the embedded samples were cut into ultra-thin sections (1 μm) and viewed under a microscope (BX53, OLYMPUS, Japan).

### 2.6 Extraction of RNA, preparation of libraries, and sequencing

Total RNA was extracted from the roots of Chinese cabbage collected at 14 dai using a TRNzol Plant RNA Extraction Kit (Tiangen) following the instructions provided by the manufacturer (Kong et al., 2021). The quality of the RNA samples OD_260/280 nm_ ≈ 1.8–2.2 and OD_260/230 nm_ ≈ 2.0 (Liu et al., 2022) was assessed using the ND-2000 Spectrophotometer from NanoDrop Technologies (Rahman et al., 2022). According to Illumina’s standard protocol, the RNA-seq libraries were prepared using 5 μg of RNA and sequenced on an Illumina Nova-seq X-Plus platform at Magigene Technology Co., Ltd. (Guangdong, China) to generate 150 bp paired-end reads. The samples used for RNA-seq analysis were labeled as R_CK, R_Pb, S_CK, and S_Pb. Here, R and S represent the resistant and susceptible cultivars, and CK and Pb represent the application of water and *P. brassicae,* respectively.

### 2.7 Transcriptome data analysis

To generate high-quality reads, the raw data was filtered using fastp (v.0.23.2) to remove the following: (1) reads containing adapters, (2) reads containing more than 10% of unknown nucleotides (N), and (3) low-quality reads containing more than 50% of low quality (Q-value ≤ 20) bases (Yang Y. et al., 2024). The clean reads were aligned to the reference genome of *B. rapa* genome version 3.0,^1^ and all of the sequencing reads were saved as FASTQ files and deposited in the NCBI Short Read Archive database (Accession number PRJNA1138284). RSEM software was employed to quantify gene expression as FPKM (Fragments Per Kilobase of exon per Million mapped fragments) values (Li and Dewey, 2011). Differentially expressed genes (DEGs) analyses were performed using DESeq2 (v1.34.0) at a threshold level of FDR < 0.05 and |log2FC| ≥1 (Liu et al., 2021). The DEGs were subsequently used for Gene Ontology (GO) functional enrichment analysis^2^ (Ashburner et al., 2000), and Kyoto Encyclopedia of Genes and Genomes (KEGG) pathway enrichment analysis^3^ (Kanehisa et al., 2021). MAPMAN was used to display gene sets on diagrams of metabolic pathways or other relevant processes (Usadel et al., 2009).

### 2.8 Validation of RNA-seq data by RT-qPCR

A quantitative RT-PCR (RT-qPCR) assay was conducted using SYBR qPCR SuperMix Plus (Novoprotein Scientific Inc., Shanghai) to evaluate the validity of root tissue RNA-Seq data. Nine DEGs were selected for amplification of RT-qPCR, and the primers were designed using NCBI primer-BLAST (Supplementary Table S1). The *GAPDH* gene was used as an internal standard. The conditions for amplification were as follows: 5 min denaturation at 95°C, followed by 35 cycles of 95°C for 15 s, 55°C for 20 s, and 72°C for 30 s. RT-qPCR was amplified for each sample with three biological and three technical replicates. The relative expression levels of genes were calculated using the 2^−ΔΔCt^ method.

## 3 Results

### 3.1 Assessment of clubroot disease incidence in Chinese cabbages

**Figure 1:**
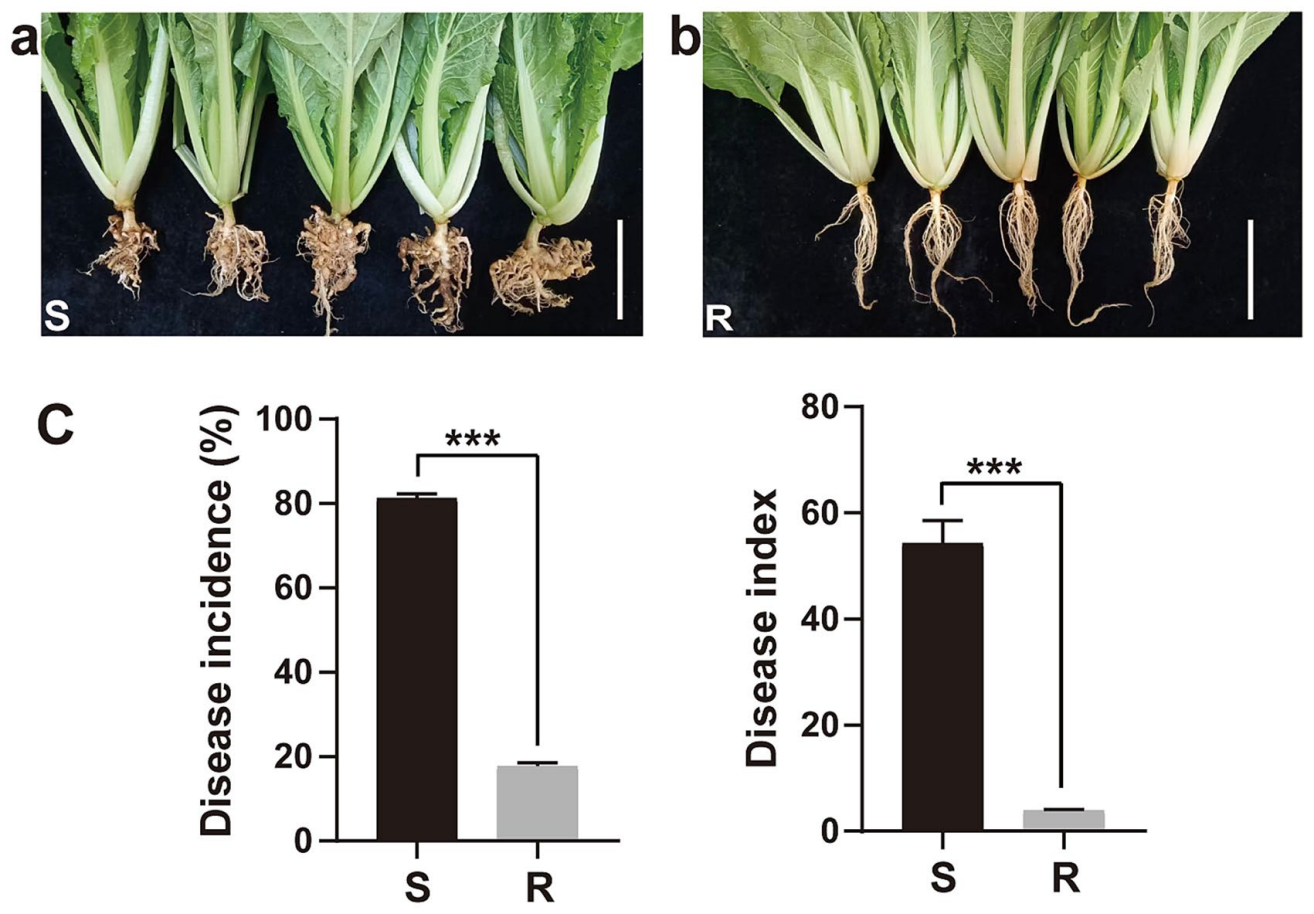
Response of two Chinese cabbage cultivars (susceptible and resistant) to clubroot pathogen *Plasmodiophora brassicae* infection. **(A)** Galls formation on the roots of susceptible cultivar 83-1 and **(B)** roots with no galls of resistant cultivar JP. White bar: 5 cm. **(C)** Disease incidence and index. R, resistant host and S, susceptible host. ***p < 0.001 indicates significant difference among treatments according to Student’s *t*-test.

The disease incidence and index of two Chinese cabbage varieties inoculated with *P. brassicae* were evaluated at 35 days after inoculation (dai) (Figure 1). Chinese cabbage cultivar 83-1 (S) was found to be highly susceptible to *P. brassicae* as compared to JP (R), and galls of different shapes and sizes were more obvious on the roots of cultivar 83-1 than JP (Figure 1A). Cultivar 83-1 exhibited a high disease incidence rate of 81.21% and a disease index of 54.28 (Figure 1C). In contrast, the cultivar JP demonstrated strong resistance to *P. brassicae* with a significantly lower disease incidence of 17.68% and a disease index of 3.93 (Figures 1B,C).

### 3.2 Microscopic observation of *Plasmodiophora brassicae* infection in different hosts

**Figure 2:**
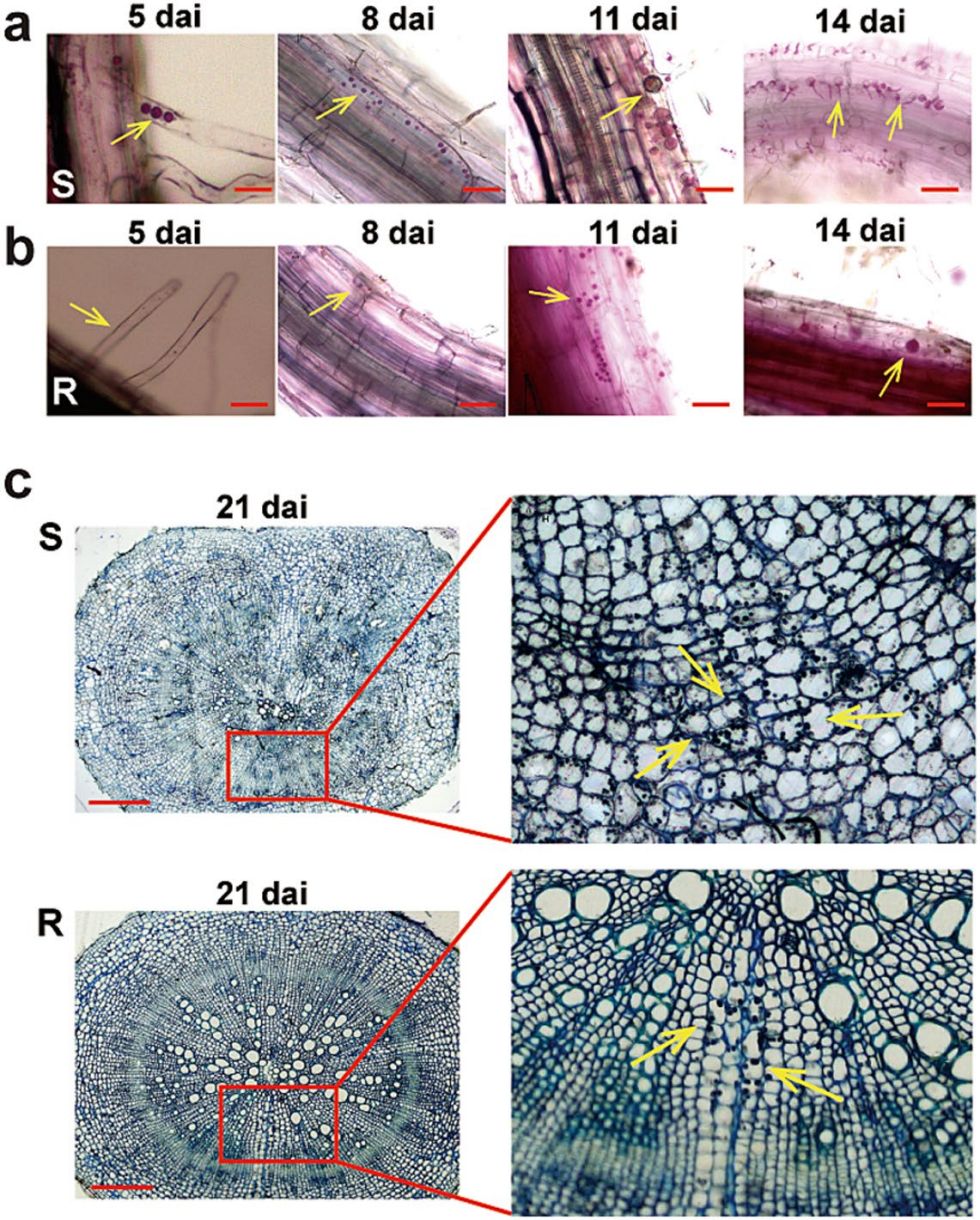
The infection process of *Plasmodiophora brassicae* in resistant and susceptible hosts. The infection process of *P. brassica* phylotype 11 in 83-1 (S; **A**) and JP (R; **B**) Chinese cabbage cultivars at 5 dai, 8 dai, 11 dai, and 14 dai. **(C)** A cross-section of the main root of both host S and R at 21 dai. The part highlighted with a red box in the lower image was further enlarged for a view in the light, and yellow arrows indicate zoosporangia. Red bar: 10 μm. R, resistant host, and S, susceptible host.

To compare the different responses of two Chinese cabbage genotypes to *P. brassicae* infection and to determine the optimal time for RNA-seq analysis, we studied the infection process of *P. brassicae*. Roots of both R and S cultivars were sampled at 5, 8, 11, and 14 dai for staining with phloxine B and microscopic observation (Figure 2). At 5 dai, a few root hairs of the S cultivar exhibited primary plasmodia, while the root hairs of the R cultivar remained unoccupied, indicating an early defense against *P. brassicae* at the primary infection (Figures 2A,B). Subsequently, at 8 dai, numerous small secondary plasmodia were observed in the cortical cells of the S cultivar. In contrast, a significantly lower number of secondary plasmodia were observed in the cortex of R cultivars roots at 8 dai. At 11 dai, the levels of secondary infection escalated in both genotypes. However, notably larger zoosporangia containing multinucleate plasmodia were observed in the cortex of the S cultivar. Interestingly, at 14 dai, a large number of zoospores began to release from the zoosporangia of the S cultivar., whereas few zoospores were observed in the R cultivar (Figures 2A,B). At 21 dai, the roots of both cultivars (S and R) were observed under the microscope. At 21 dai, small galls were visible in the roots of the S cultivar, and in the cross-section of the primary root, the distortion organization of tissues was visible. The phloem and xylem cells were numerous and minor, and most of the cells and intercellular spaces were filled with sporangia. However, only a few cells in the cortex of the R variety were filled with sporangia, and there was no evident distortion in cortex cells (Figure 2C). No visible galls could also be observed in the root of the R cultivar. Therefore, we assume that 14 days may be a critical time point for the development of *P. brassicae* infection in hosts with different resistance levels.

### 3.3 Transcriptome sequencing and quality assessment

**Figure 3:**
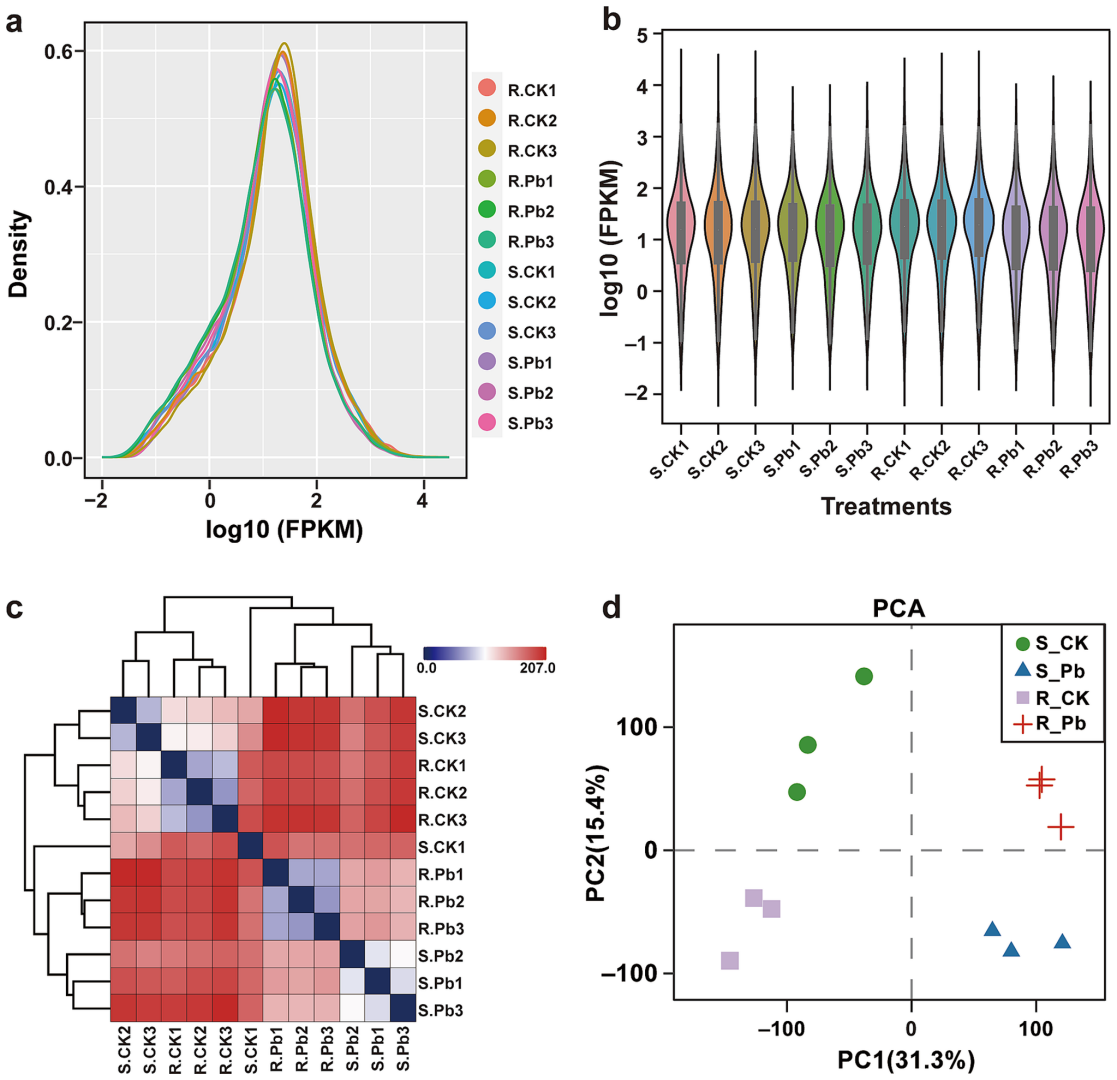
The data quality of RNA-seq in all samples. **(A)** FPKM density for each sample, the horizontal axis represents different samples, and the vertical axis represents the FPKM for samples. **(B)** The violin plot shows the sample expression levels; each color in the figure represents a sample. **(C)** Distance heatmap for correlation analysis of the samples. On the right side of the figure is the sample name, and on the left and upper sides are the sample clustering situation. **(D)** PCA analysis for sample clustering. R, resistant host; S, susceptible host; and Pb, application of *P. brassicae*.

The analysis aims to identify transcriptome differences between S and R cultivars in response to *P. brassicae* infection at 14 dai by comparing the infected samples to their corresponding controls, each with three biological replicates. After sequencing, 41 ~ 58 million raw reads were obtained for each sample, with 6.54 Gb clean base on average (Supplementary Table S2). The GC content of all samples ranges from 45.81 to 48.73%, and the Q20 (%) were all greater than 97.5%, indicating a high accuracy of base recognition. Approximately 77.27 to 93.63% of high-quality reads in each sample were successfully mapped to the reference genome of Chinese cabbage (*Brassica rapa* CAAS_Brap_v3.01). First, we assessed the FPKM distribution density and FPKM expression levels for each sample under control and treatments. The results of sequencing quality and gene expression levels for all samples revealed that each sample yielded the same coverage depth and read levels (Figures 3A,B). A correlation heatmap revealed a strong correlation among the samples within a treatment (Figure 3C). Additionally, the principal component analysis revealed a 46.7% variation in the transcripts of R and S cultivars under different treatments (Figure 3D). Thus, the results of genome alignment indicate that the sequencing quality is satisfactory, meets the data quantity and experimental requirement standards, and is suitable for subsequent analyses.

### 3.4 Identification of differentially expressed genes

**Figure 4:**
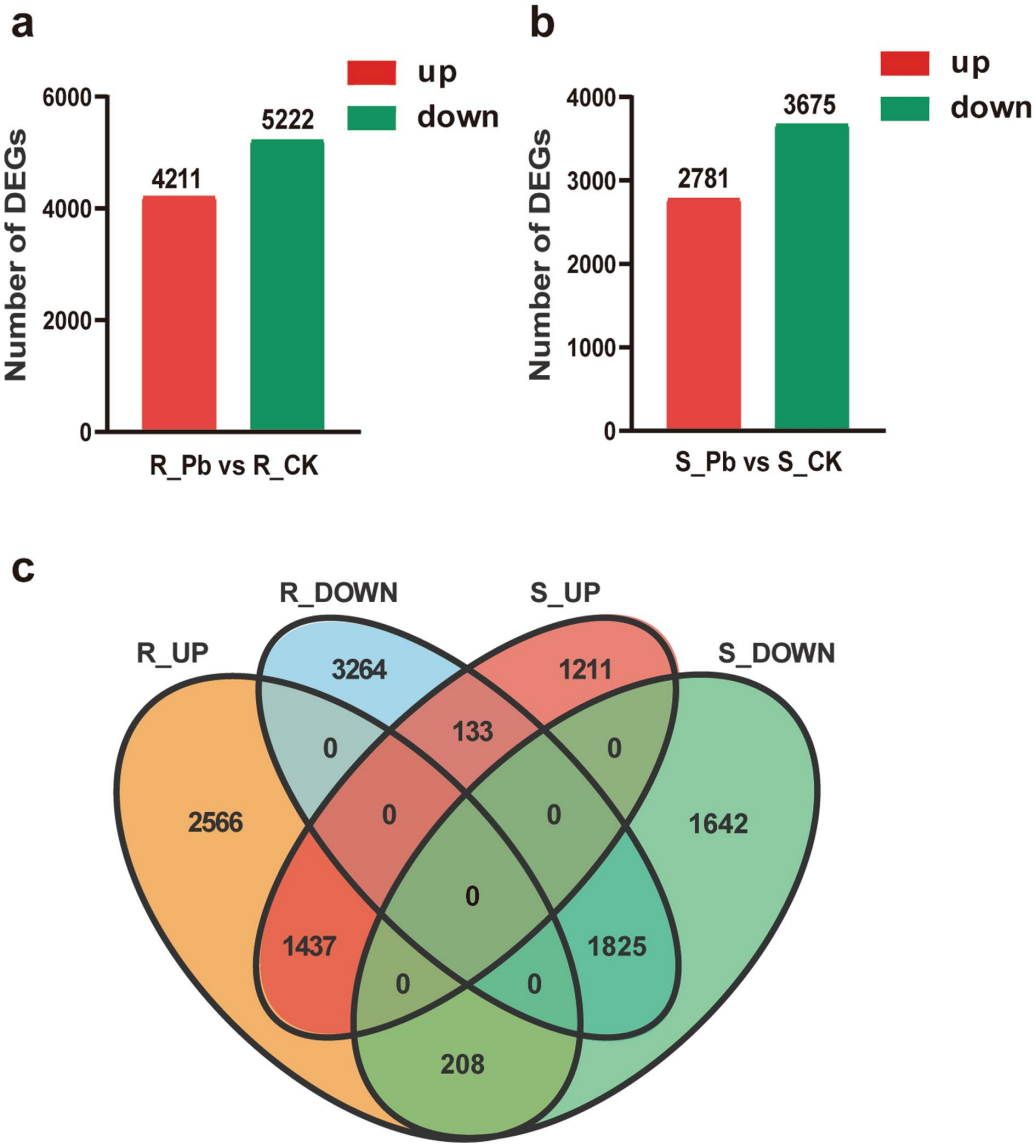
Histogram and Venn diagram of differentially expressed genes (DEGs) in the Chinese cabbage cultivars after *P. brassicae* inoculation. **(A)** DEGs between the resistant cultivar JP (R) control plants and cultivar JP plants infected with pathotype 11 of *P. brassicae* at 14 dai. **(B)** DEGs between the susceptible cultivar 83-1 (S) control plants and cultivar 83-1 plants infected with pathotype 11 of *P. brassicae* at 14 dai. **(C)** Venn diagram of up-regulated and down-regulated DEGs in the R and S groups, respectively. R, resistant host; S, susceptible host; and Pb, application of *P. brassicae*.

**Figure 5:**
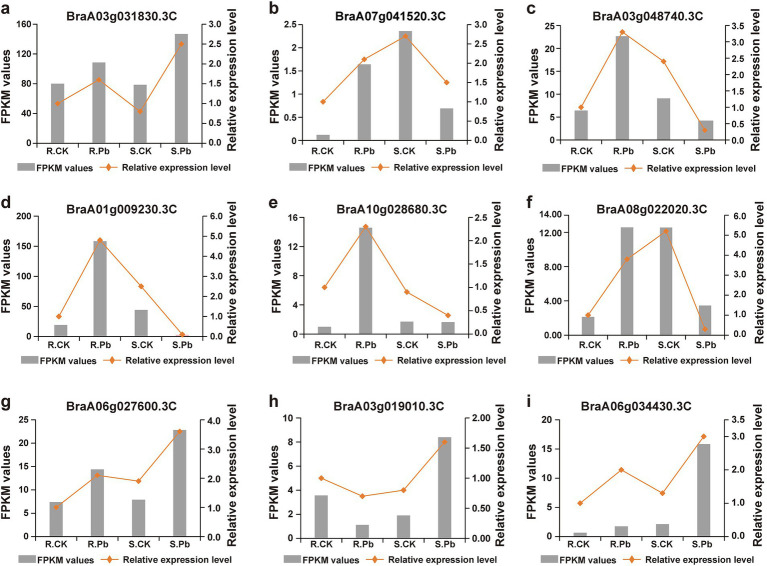
Validation of RNA-seq data by RT-qPCR. **(A–I)** DEGs were randomly selected for amplification of RT-qPCR for validation of RNA-seq data.

To compare the differential response to *P. brassicae* infection between the two cultivars of Chinese cabbage (JP and 83-1), the transcripts of each infected library were compared with transcripts of control libraries for each Chinese cabbage cultivar. In the R cultivar (R_Pb vs. R_CK), a total of 9,433 DEGs were significantly expressed, in which 4,211 DEGs were up-regulated and 5,222 were down-regulated (Figure 4A). In the S cultivar (S_Pb vs. S_CK), 6,456 DEGs were identified, with 2,781 DEGs up-regulated and 3,675 DEGs down-regulated (Figure 4B). The observation of fewer DEGs in the S cultivar than in the R cultivar indicates that a greater number of DEGs in the R host participated in the defense against *P. brassicae* infection. In order to gain a deeper insight into the defense molecular mechanisms in *B. rapa*, DEGs were divided into four groups: DEGs up-regulated in both R and S (co-up), DEGs down-regulated in both R and S (co_down), DEGs up-regulated in R but down-regulated in S (R_UP/S_DOWN), and DEGs down-regulated in R but up-regulated in S (R_DOWN/S_UP). From the Venn chart (Figure 4C), it was observed that there were 1,437 and 1825 DEGs that exhibited co-up and co-down regulation in the R and S groups, respectively. A total of 208 DEGs were found to be up-and down-regulated in the R_UP/S_DOWN group (Figure 4C; Supplementary Table S3). Genes like calcineurin B-like protein (*BraA01g009230.3C*), cellulose synthase A catalytic (*BraA03g004030.3C*), polyamine oxidase (*BraA03g005860.3C*), and 9-cis-epoxycarotenoid dioxygenase (*BraA07g041520.3C*) are associated with plant resistance based on previous studies (Jung et al., 2017; Liu et al., 2018; Burris et al., 2021; Wang et al., 2021; Benkő et al., 2023), and are therefore supposed to contribute to host resistance. On the contrary, 133 DEGs were down-regulated in R but up-regulated in S (R_DOWN/S_UP), including growth-regulating factor (BraA03g019010.3C), auxin efflux carrier component (*BraA07g029730.3C*) (Figure 4C; Supplementary Table S4), which are considered to be associated with susceptibility. To confirm the reliability of the transcriptome sequencing, nine genes were randomly selected to perform RT-qPCR. The relative expression levels of these genes detected by RT-qPCR were consistent with the FPKM values from RNA-seq (Figure 5). These results suggest the reliability of the RNA-Seq data used in the study.

### 3.5 Functional annotation and enrichment analysis of DEGs

**Figure 6:**
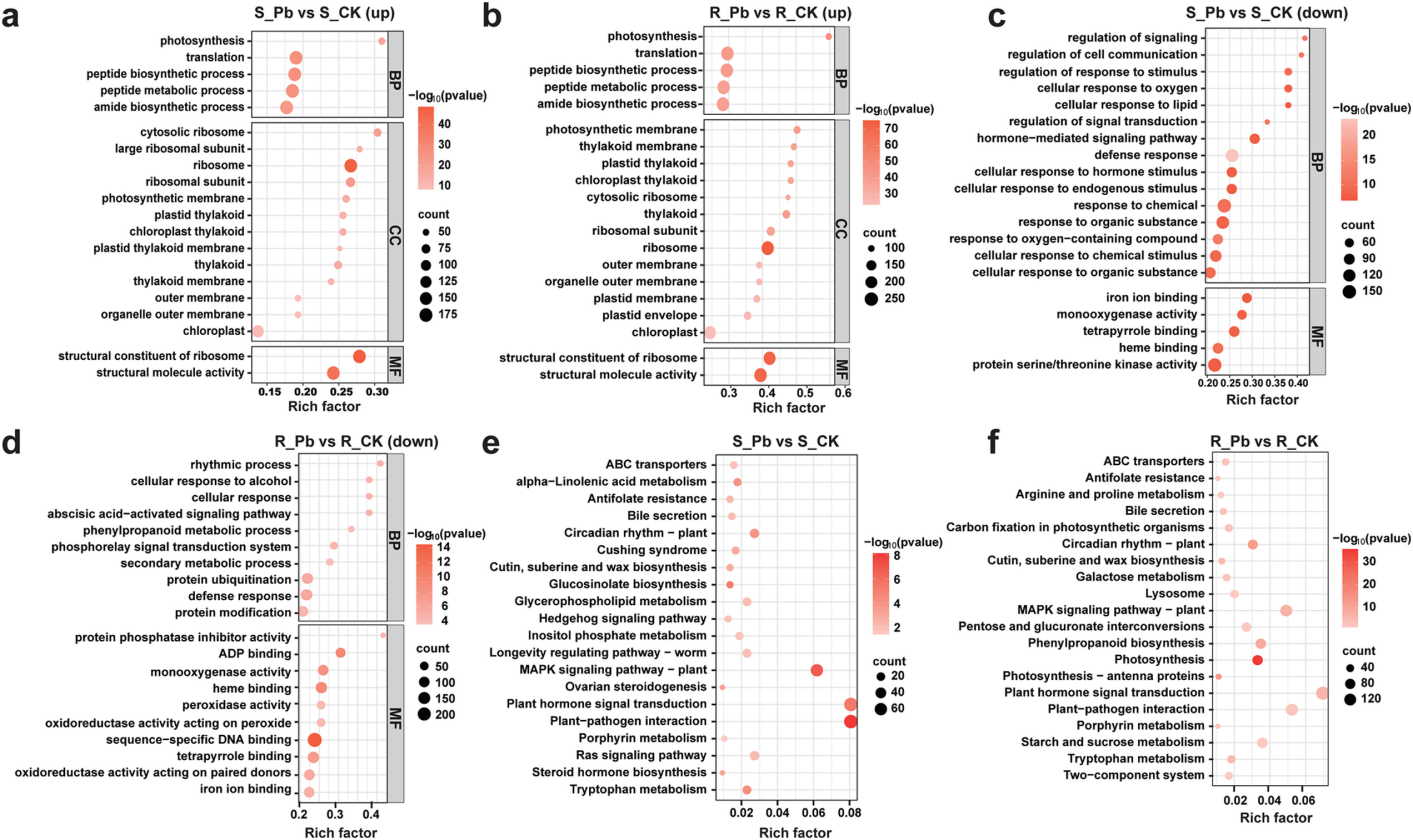
Bubble plots of GO and KEGG pathway enrichment analysis. **(A)** GO enrichment of up-regulated genes in S_Pb vs. S_CK; **(B)** GO enrichment of up-regulated genes in R_Pb vs. R_CK; **(C)** GO enrichment of down-regulated genes in S_Pb vs. S_CK; **(D)** GO enrichment of down-regulated genes in R_Pb vs. R_CK; **(E)** KEGG enrichment of DEGs in S_Pb vs. S_CK; **(F)** KEGG enrichment of DEGs in R_Pb vs. S_CK. The horizontal axis represents the rich factor, while the vertical axis represents the enriched pathway name. The color scale indicates different threshold levels of the *p*-value, and the dot size indicates the number of genes corresponding to each pathway. R, resistant host; S, susceptible host; and Pb, application of *P. brassicae*.

GO functional annotation was performed in a groupwise comparison between the control samples and the corresponding infected groups. All of the DEGs were assigned to three GO classes: biological process (BP), cellular component (CC), and molecular function (MF). In the R group, BP, CC, and MF were further classified into 16, 2, and 16 subclasses, respectively. Among all the subclasses, cellular anatomical entity (GO:0110165), binding (GO:0005488), and catalytic activity (GO:0003824) were the top three highly expressed subcategories in the R group. In comparison, all DEGs in the S group were classified into 16, 2, and 14 subclasses, respectively (Supplementary Figure S1). It is worth noting that compared to the S group, more up-regulated genes in the R group were annotated into cellular and metabolic processes, indicating that these genes may play a key role in disease resistance. To better understand the GO functions of DEGs, we conducted enrichment analysis on two groups of up-regulated and down-regulated genes, respectively. The top 20 GO terms with significantly enriched (*p*-value < 0.05) were selected for display and analysis. The top five GO terms of up-regulated DEGs from S group (S_Pb vs. S_CK) were peptide metabolic process (GO:0006518), ribosome (GO:0005840), translation (GO:0006412), peptide biosynthetic process (GO:0043043), and amide biosynthetic process (GO:0043604) (Figure 6A), while the top five GO terms of up-regulated DEGs in the R group (R_Pb vs. R_CK), were structural molecule activity (GO:0005198), amide biosynthetic process (GO:0043604), peptide metabolic process (GO:0006518), peptide biosynthetic process (GO:0043043), and ribosome (GO:0005840) (Figure 6B). It can be seen that the up-regulated DEGs in both groups were enriched in the similar GO terms. It may indicate that the functional types of DEGs caused by the infection of *P. brassicae* on the two hosts are similar, but each GO term in the R group has enriched more DEGs than the S group. However, the top five GO terms of the down-regulated DEGs in S group were response to chemical (GO:0042221), protein serine/threonine kinase activity (GO:0071900), defense response (GO:0006952), response to the organic substance (GO:0010033) and cellular response to chemical stimulus (GO:0070887) (Figure 6C), and top five GO terms of down-regulated DEGs in the R group were sequence-specific DNA binding (GO:0043565), defense response (GO:0006952), heme binding (GO:0020037), tetrapyrrole binding (GO:0046906), and protein modification (GO:0070647) (Figure 6D). These results indicates that the highest percentage of down regulated genes in both groups were mapped to different GO terms with different functions. In addition, the R group showed more downregulated DEGs related to molecular function, while in the S group, more downregulated DEGs were assigned to biological processes. Perhaps these differences have led to differences in resistance to *P. brassicae*.

### 3.6 Insights into the enriched KEGG pathways

To investigate the enriched pathway for DEGs further, KEGG pathway enrichment analysis was performed on the DEGs of two groups (R_Pb vs. R_CK and S_Pb vs. S_CK). The results demonstrated that the top three enriched pathways in both R and S groups were plant hormone signaling transduction (ko04075), plant-pathogen interaction (ko04626), and MAPK signaling pathway in plants (ko04016) (Figures 6E,F; Supplementary Tables S5, S6). However, more genes were enriched in the R group within each pathway, and more genes were up-regulated. It is worth noting that Phenylpropanoid biosynthesis (ko00940) was enriched in the R group but not enriched in the S group (Supplementary Table S6). Some genes in this pathway such as 4CL (*BraA03g039680.3C*, *BraA03g039690.3C*), PAL (*BraA04g006280.3C*, *BraA04g015350.3C*) and CCR (*BraA03g055800.3C*) are key enzymes for the synthesis of lignin, which were up-regulated (Supplementary Table S7). These results indicated that the R host might strengthen the cell wall structure by increasing the lignification of the cell wall, limiting the space for the proliferation of *P. brassicae* in the root, thereby inhibiting the formation of clubroot. Therefore, we assumed that phenylpropanoid biosynthesis is an important pathway for the R host to resist the infection of *P. brassicae*.

### 3.7 Overview of biotic stress-related pathways

**Figure 7:**
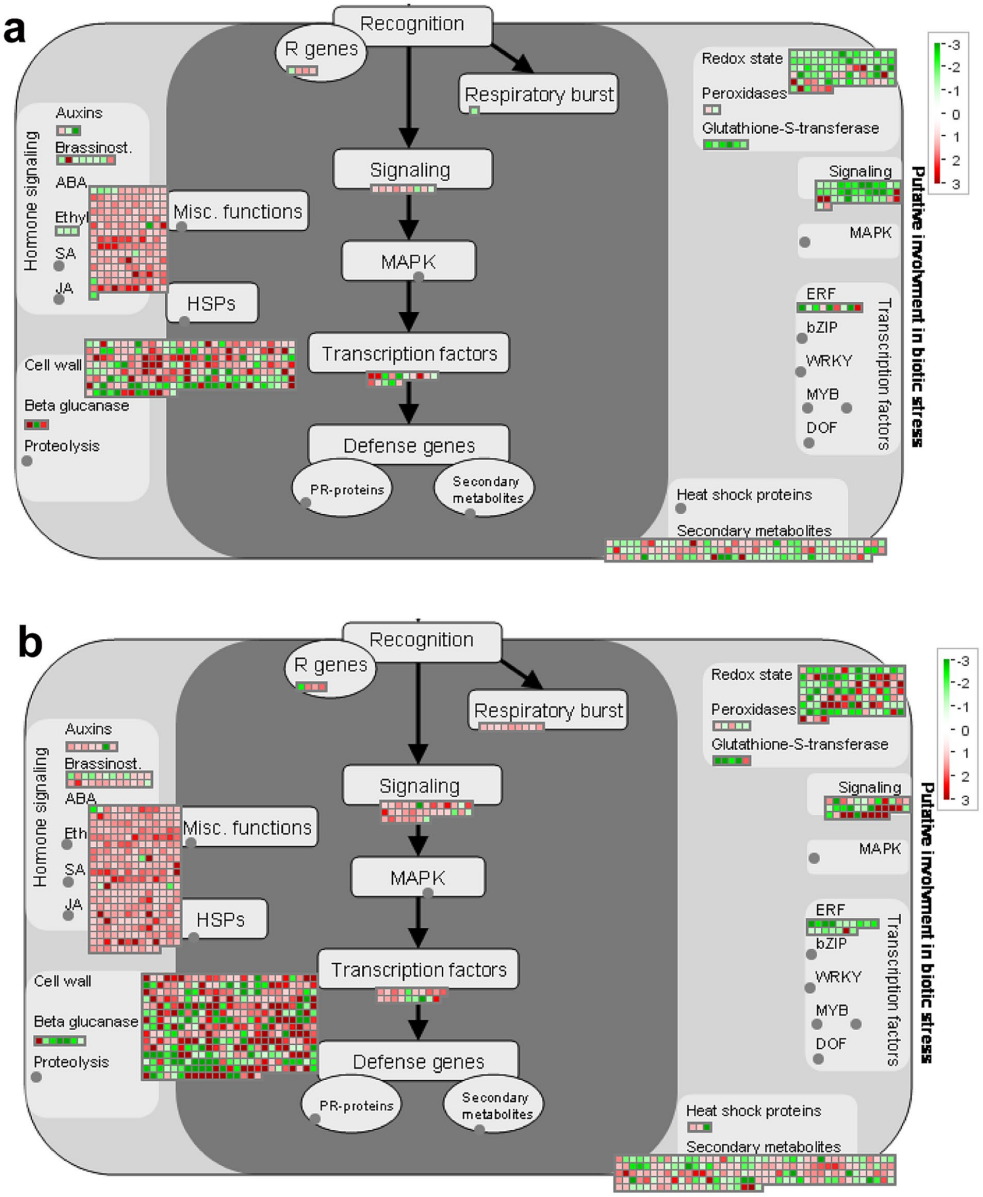
Distribution of DEGs involved in the biotic stress response in two groups of hosts after inoculation with *P. brassicae*. **(A)** S_Pb VS S_CK, **(B)** R_Pb VS R_CK. Red boxes mean up-regulated genes, and green mean down-regulated genes. The regulation of genes is based on log2 fold change. R, resistant host; S, susceptible host; and Pb, application of *P. brassicae*.

To further analyze the function of DEGs, we use MAPMAN software to visualize the regulation of genes in various biotic correlated responses. According to MAPMAN analysis, 724 DEGs in the S group and 1,130 DEGs in the R group were mapped in the biotic stress pathway, with similar categories in the two groups. The pathway overview included five parts: hormone signaling, cell wall-related genes, secondary metabolism, redox state, and signal process (Figure 7).

### 3.8 Genes related to respiratory burst

Respiratory burst (RB) is a rapid increase in the production of reactive oxygen species (ROS) during the phagocytosis of microbes. In the R plant, a total of nine DEGs (*BraA08g003230.3C*, *BraA02g008410.3C*, *BraA10g020620.3C*, *BraA04g021170.3C*, *BraA05g010320.3C*, *BraA05g014630.3C*, *BraA06g043160.3C*, *BraA07g039280.3C*, *BraA09g020440.3C*) were assigned to respiratory burst, and they were all up-regulated (Figure 7B). In contrast, in the S plant, only one gene (BraA02g008420.3c) was involved in this term and was down-regulated (Figure 7A; Supplementary Table S8). This result suggests that the respiratory burst pathway may play an important role in resistance for R hosts to the *P. brassica* infection.

### 3.9 Genes related to hormone signaling

Plant hormones play an important role in the plant-pathogen interaction system, especially during the process of root swelling caused by the infection of *P. brassicae*. In our study, the results showed that the expression of ABA signaling genes changed significantly in both hosts after being infected by *P. brassicae* (Supplementary Table S9), 164 DEGs (157 up-regulated and 7 down-regulated) in the S group and 268 DEGs (263 up-regulated and 5 down-regulated) in R group. Forty-eight DEGs (38 up-regulated and 10 down-regulated) and 71 DEGs (61 up-regulated and 10 down-regulated) related to the gibberellin (GA) signaling pathway were induced in S and R, respectively. DEGs involved in Indole-acetic acid (IAA), Brassinosteroid (BA) and cytokinin signaling pathways were down-regulated in the S host but were up-regulated in the R host. Only three DEGs were classified in the ET category, and all were down-regulated in S. In contrast, no DEGs were classified in this category in R (Supplementary Figure S2).

### 3.10 Genes related to cell wall

The cell wall is the first barrier for pathogen invasion; the changes in cell wall-related genes at the transcriptional level could partially reflect the host’s response to pathogens. According to the analysis of MAPMAN, it was evident that a large proportion of the DEGs were involved in the category of cell wall. The variation in gene expression levels in S and R root tissue after inoculating *P. brassicae* can be intuitively observed in the heat map (Supplementary Figure S3). The regulation of genes such as cellulose synthase CESA (*BraA03g002020.3*, *BraA06g043400.3*, *BraA03g004030.3*), COBRA-like protein COBL (*BraA02g006110.3C*), cellulose synthase-like protein CSLD (*BraA10g022900.3*), galacturonosyltransferase GATL (*BraA07g034960.3*) and xyloglucan glycosyltransferase MUR (*BraA07g000460.3*) involved in cell wall synthesis and lignification are up-regulated in the R root but down-regulated in the S root after being infected by *P. brassicae* (Supplementary Figure S3A). DEGs encoding for EXPA (*BraA03g020980.3*, *BraA01g019010.3*, *BraA03g054290.3*), FLA (*BraA07g024430.3*) and PMEs (*BraA01g016010.3*, *BraA05g000370.3C*, *BraA06g011010.3C*) involved in cell wall modification, which are responsible for cell wall-loosening processes, were up-regulated in S and down-regulated in R. The xyloglucan endotransglucosylase (*XTHs*) were up-regulated after infection in both hosts; however, the expression levels of these genes in the R root are lower than those in the S root (Supplementary Figure S3B). Intriguingly, genes encoding hydrolytic enzymes (*BGALs*, *MANs* and *QRTs*) of cell wall degradation in both hosts had a similar tendency after inoculating with *P. brassicae* (Supplementary Figure S3C). This indicates that during the process of clubroot establishment, infection of *P. brassicae* induced genes involved in cell division and expansion, rather than hydrolysis degradation of structural cell wall components.

### 3.11 Genes related to secondary metabolites

**Figure 8:**
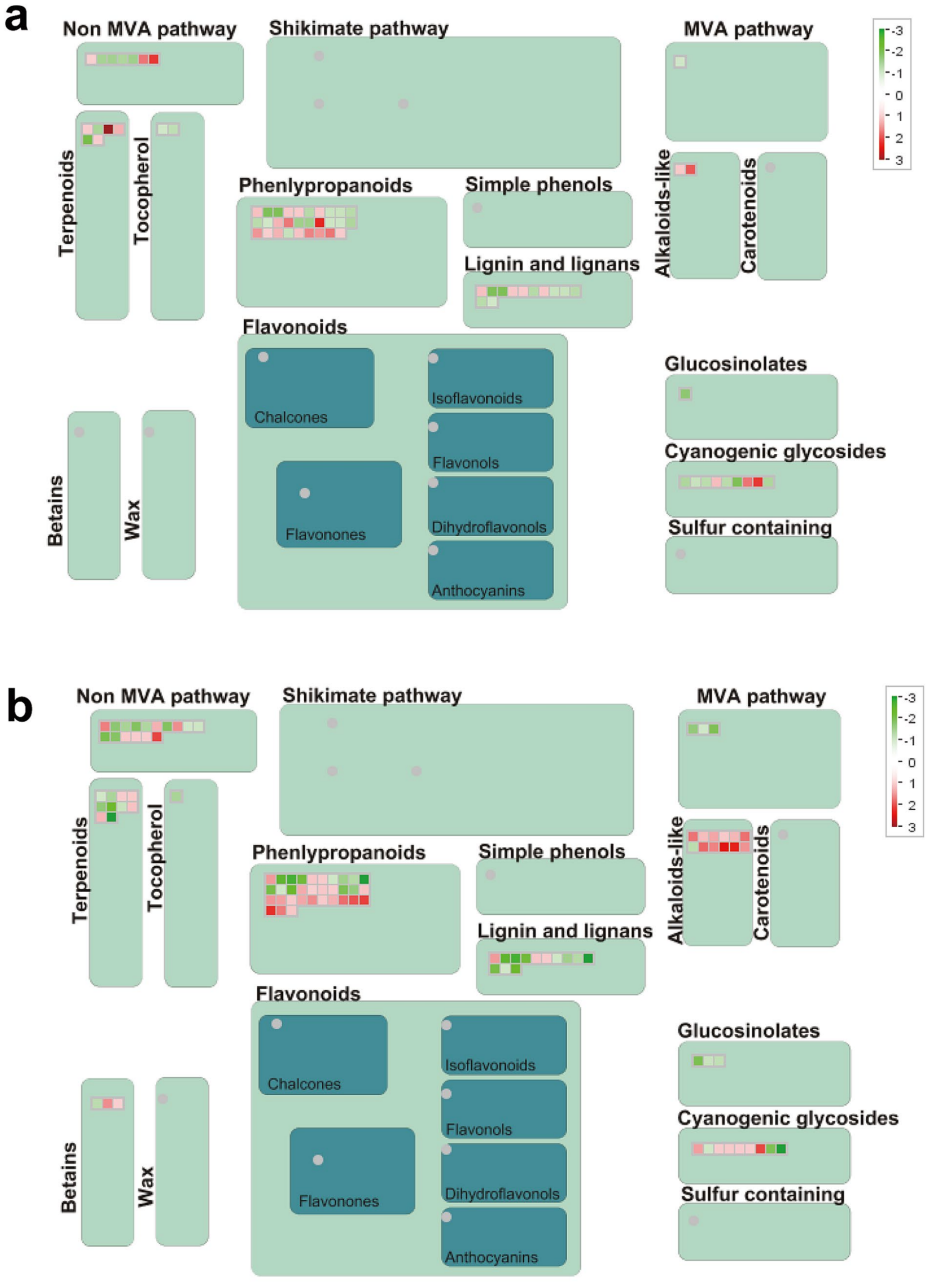
Secondary metabolism pathway analyses of DEGs. **(A)** S_Pb VS S_CK, **(B)** R_Pb VS R_CK. Red boxes mean up-regulated genes, and green mean down-regulated genes. R, resistant host; S, susceptible host; and Pb, application of *P. brassicae*.

The alteration in gene expression of secondary metabolites exhibits notably more pronounced changes in R than S (Figures 8A,B). In S, there were more down-regulated genes (30 up-regulated and 39 down-regulated). Meanwhile, R had more up-regulated genes (54 up-regulated and 49 down-regulated). Intriguingly, over close observation of the secondary metabolism pathway, it becomes evident that the DEGs related to the biosynthesis of phenylpropanoids, lignin and cyanogenic glycosides were down-regulated in S (Figure 8A; Supplementary Table S10-1). However, the DEGs related to the biosynthesis of phenylpropanoids, cyanogenic glycosides and alkaloids were up-regulated in R (Figure 8B; Supplementary Table S10-2). The discrepancy in the expression of these genes suggests that the secondary metabolites pathway may play roles in gall’s development.

## 4 Discussion

Clubroot is considered a devastating disease of cruciferous crops, causing severe yield losses worldwide (Yang X. et al., 2024). *P. brassicae* is a soilborne obligate biotrophic pathogen that cannot be cultured *in vitro*, making it difficult to decipher the molecular mechanisms of *P. brassicae*-Brassica interactions. Observing and comparing the host’s response to the infection of *P. brassicae* is the only available method of indirectly analyzing the interaction mechanism between pathogen and their host. With the development of RNA-seq technology, conducting comparative analyses of the transcriptional differences between resistant and susceptible varieties can provide molecular insight into the invasion and defense strategies of *P. brassicae* and their hosts. The differential response of resistant and susceptible hosts was frequently noted by many researchers who are dedicated to exploring the molecular mechanism of host resistance (Wang et al., 2019; Galindo-González et al., 2020; Zhou et al., 2020). The present study investigates the interactions between specific pathotypes and host genotypes, aiming to dissect the constitutive or induced defenses mechanism.

The life cycle of *P. brassicae* is closely related to the development of galls in the root system of host plants. The size of the gall is considered an indicator of the severity of clubroot and a standard for classifying a host as resistant or susceptible to clubroot. However, previous studies have shown that gall formation is not essential for the completion of the *P. brassicae* life cycle (Malinowski et al., 2012). From the process of *P. brassicae* infection, it was recorded that the highest levels of secondary infection occur at 14 dai in the S host with a large amount of zoospore released, causing a new round of infection and finally leading to the formation of galls at the 21dai, is the indication of complete collapse of host’s immune system. However, no visible galls were observed in the root system of the R host, although cross-sections through the hypocotyl of infected R plants at 21 DAI showed that zoosporangia presented in the phloem and xylem. However, the number of zoosporangia in the xylem is significantly less than in the phloem. Moreover, it was noticed that the structure of root tissue remains intact and not disturbed by the parasite. This result indicates that the R host is not immune to *P. brassicae* invasion, but developed a strategy to reduce the infection rate and inhibit their large-scale reproduction within the stele. The resistance mechanism of this strategy is different from the *WTS* gene. *WTS* is a broad-spectrum clubroot resistance gene discovered and characterized by Wang and his colleagues in *Arabidopsis.* Upon infection by the pathogen, it can be activated in the pericycle to prevent the pathogen colonization in the stele (Wang et al., 2023).

During infection, the most destructive aspect to the host is significant cell enlargement and proliferation. These symptoms indicate the involvement of the plant hormones changes in disease development, mainly auxin and cytokinin. Several reports have illustrated the potential role of auxin and cytokinin during the different and rather late stages of *P. brassicae* infection (Robin et al., 2020; Bíbová et al., 2023). During the early stages of infection, the initial infection leads to an increase in the total auxin pool, resulting in a temporary stimulation of plant growth (Devos et al., 2005; Xu et al., 2016). In contrast, in the later stage, the levels of IAA detected in infected roots were lower than those in control roots (Ludwig-Müller and Schuller, 2008). In the present study, more DEGs related to IAA and cytokinin pathway were up-regulated in R than in S host. The results seem contradictory, and this might be due to the different pathotypes of *P. brassicae* being used to infect the host with different genotypes, and hormone changes may be transient (Ciaghi et al., 2019).

Jasmonic acid (JA), salicylic acid (SA), ethylene (ET), and abscisic acid (ABA) are pivotal plant hormones known for their significant role in mediating responses to both abiotic and biotic stresses. Multiple lines of evidence indicate that JA and SA-mediated signaling pathways are involved in the regulating defense response to *P. brassicae* (Fu et al., 2019; Wang et al., 2019; Zhou et al., 2020; Wei X. et al., 2021). In the early infection of *P. brassicae,* genes involved in the JA signaling pathway were inhibited in *B. rapa* (contained *CRb* gene) but activated at the late stage of infection (Jia et al., 2017). The same results were found in *Arabidopsis thaliana* (Divi et al., 2010) and *B. oleracea* (Zhang et al., 2016). Other results from the comparative transcriptome analysis of Rutabaga cultivars showed that JA does not seem to be the core defense of resistant varieties but may be an activated mechanism in the interaction between susceptible varieties and pathogens (Wang et al., 2019). However, SA has been reported to have a suppressive effect on clubroot and treatment with exogenous salicylic acid reduced the development of clubroot in *A. thaliana* and *B. napus* (Lovelock et al., 2012; Chen T. et al., 2016). The role of the SA signaling pathway in *B. rapa* resistance to *P. brassicae* infection was demonstrated (Chen J. et al., 2016). Zhang et al. (2016) also reported that the TGA4-NPR1 interaction in the SA-dependent pathway may contribute to disease resistance against *P. brassicae* in B2013 (a clubroot-resistant wild cabbage) (Zhang et al., 2016). In the present study, no genes were mapped to the JA and SA pathways through MAPMAN analysis. However, when we analyzed the genes related to JA and SA in plant hormone signaling transduction from KEGG enriched pathway, including *JAR1*, *COI-1*, *JAZ*, *MYC2*, *NPR1*, *TGA*, and *PR1*, we found that in the R group, most JA related genes were up-regulated while most SA related genes were down-regulated. More interestingly, the gene expression in S group was opposite to that in group R (Supplementary Table S11). Genes encoding jasmonate ZIM domain (*JAZ*), which involved in the JA signaling pathway were all up-regulated in R group, while 21out of 22 genes were down-regulated in S group. These results are consistent with the findings of Zhang et al. (2016) and Li et al. (2020), who found that *JAZs* were up-regulated in R-line and inhibiting the JA signaling pathway to improve the CR. Further detailed analysis of hormonal involvement is warranted due to the complexity of temporal control of hormones during the various phases of the disease.

In the discussion, we recognize the established role of the regulatory function of ABA in plant response to abiotic stress such as drought, salt, and cold. Recent studies have also shed light on regulatory responses to biological stress by antagonizing ET and JA (Song and Wu, 2024). Devos and colleagues reported for the first time that ABA played a pivotal role in the development of clubroot in *B. rapa* roots (Devos et al., 2005). At 21 DAI, the ABA content of infected roots in Chinese cabbage significantly increased compared to the control roots and is consistent with the gall’s formation. Analyzing the transcriptome data of *B. rapa* revealed that the changes in gene expression levels involved in ABA signaling pathway, were in line with the measured ABA content (Wei X. et al., 2021). Therefore, we speculate that the high expression of ABA-related genes in our study may help Chinese cabbage tolerate water limitation conditions caused by *P. brassicae* infection. As the role of ABA in plant defense is very complex and differs in different plant-pathogen interactions, further research will be needed to confirm ABA’s role in the Clubroot process.

Secondary metabolites are trace organic substances produced by plants and can serve as chemical weapons against herbivores, insects, and other biological stressors (Das Laha et al., 2024). Phenylpropanoids, flavonoids, and phenols are the primary secondary metabolites that act on pathogens through enzyme inhibition, DNA alkylation, and reproductive systems (Anjali et al., 2023). Some flavonoids, like flavanone and isoflavone, inhibit the growth and infection of oomycetes pathogens. Three flavanones were isolated from *Kunzea robusta*, which significantly reduced the zoospore germination of *Phytopthora agathidicida* (Lawrence et al., 2019). *Cascalote phenolics* have *in vitro* fungistatic activity against *C. lindemuthianum,* which causes anthracnose disease in common beans (*Phaseolus vulgaris*) (Veloz-García et al., 2010). Alkaloids, such as caffeine, atropine, and quinine, help plants develop complex defense systems against invasive pathogens (Erharuyi et al., 2014). Bruceine-D is an alkaloid found in *Brassica javanica* extract, which has inhibitory activity against PVY, CMV, and TMV (Shen et al., 2015). Cyanogenic glycosides are essential secondary metabolites with biological activity and are involved in regulating oxidative stress and play an essential role in plant defense response (Gleadow and Møller, 2014; Chen et al., 2022). In the present study, we found that DEGs in the secondary metabolites, including phenylpropanoids, alkaloids and cyanogenic glycosides, were significantly up-regulated in the R plant. In contrast, in the S plant, the regulation was just the opposite. Therefore, we speculate that in our experiment, the R variety of Chinese cabbage inhibits the proliferation of *P. brassicae* in the roots through the synthesis of functional secondary metabolites.

Transcriptome analysis of *B. rapa* infected by *P. brassicae* showed that genes associated with PR, hormone signaling (JA, SA and ET), RBOH proteins, calcium ion influx and cell-wall modification played important roles in the interaction between *B. rapa* and *P. brassicae* at the early stages of infection (Chen J. et al., 2016). Another comparative transcriptome analysis revealed that genes associated with auxin, PR proteins, disease resistance proteins, oxidative stress, and WRKY and MYB transcription factors play important roles in R-line Chinese cabbage against *P. brassicae* pathotype 4 (Yuan et al., 2021). In this study, we identified 9 genes associated with Ca^2+^ signal transduction, such as calcineurin B-like protein, calcium uniporter protein, calcium-dependent protein kinase and calmodulin-binding protein were up-regulated in R, but down-regulated in S. Meanwhile, more genes involved in respiratory burst and cell wall-related were up-regulated in R. Still, no genes related to jasmonic acid, salicylic acid and ethylene were identified. It is evident that there are differences in the resistance mechanisms of R-line Chinese cabbage varieties to different physiological races. These differences may be due to the inconsistent genetic backgrounds of different host varieties and physiological races, resulting in differences in the expression levels of host genes. In addition, the sampling time is crucial, as it is consistent with the progression of the disease, which can more accurately reflect the host’s resistance mechanism to *P. brassicae*. Understanding the resistance mechanisms of Chinese cabbage to different physiological races would be beneficial for guiding the breeding of disease-resistant varieties.

## 5 Conclusion

In conclusion, in this study, we first investigated molecular mechanisms of resistance against *Plasmodiophora brassicae* pathotype 11 infection in Chinese cabbage. Comparative transcriptome analysis of resistant (R) and susceptible (S) cultivars revealed a greater number of DEGs in the R (JP) cultivar compared to the S (83-1) one, and the up-regulated DEGs in R were involved in critical defense pathways, including respiratory burst, hormone signaling, and secondary metabolism. Our speculation revolves around the potential resistant mechanism of this variety, which inhibits the proliferation of *P. brassicae* in the roots via secondary metabolites, cell wall, and ROS and also regulates physiological mechanisms mediated by plant hormones such as ABA to adapt to adverse environmental conditions such as water scarcity induced by the pathogen. Therefore, our results reveal a molecular mechanism that differs from previously published results elsewhere. However, future research will be carried out to elucidate the specific roles of identified DEGs in the resistance response and explore potential interactions between different defense pathways. Additionally, functional validation of key genes through genetic manipulation or other approaches would provide more definitive evidence of their roles in resistance.

## Data Availability

The datasets presented in this study can be found in online repositories. The names of the repository/repositories and accession number(s) can be found below: https://www.ncbi.nlm.nih.gov/, PRJNA1138284.

## Footnotes

^1^ http://brassicadb.cn

^2^ https://github.com/tanghaibao/Goatools

^3^ http://kobas.cbi.pku.edu.cn/home.do
